# Robust detection of heartbeats using association models from blood pressure and EEG signals

**DOI:** 10.1186/s12938-016-0122-0

**Published:** 2016-01-15

**Authors:** Taegyun Jeon, Jongmin Yu, Witold Pedrycz, Moongu Jeon, Boreom Lee, Byeongcheol Lee

**Affiliations:** School of Information and Communications, Gwangju Institute of Science and Technology, 261 Cheomdan-Gwagiro, Buk-gu, Gwangju, Republic of Korea; Department of Electrical and Computer Engineering, University of Alberta, Edmonton, T6R 2V4 Alberta, Canada; Systems Research Institute, Polish Academy of Sciences, Warsaw, Poland; Department of Electrical and Computer Engineering Faculty of Engineering, King Abdulaziz University Jeddah, Jeddah, Saudi Arabia; Department of Medical System Engineering (DMSE) and School of Mechatronics, Gwangju Institute of Science and Technology, 261 Cheomdan-Gwagiro, Buk-gu, Gwangju, Republic of Korea

**Keywords:** Blood pressure, Electrocardiogram, Electroencephalogram, Estimation of regular intervals, Heartbeats detection, Multimodal fusion, QRS detection, Signal quality index

## Abstract

**Backgrounds:**

The heartbeat is fundamental cardiac activity which is straightforwardly detected with a variety of measurement techniques for analyzing physiological signals. Unfortunately, unexpected noise or contaminated signals can distort or cut out electrocardiogram (ECG) signals in practice, misleading the heartbeat detectors to report a false heart rate or suspend itself for a considerable length of time in the worst case. To deal with the problem of unreliable heartbeat detection, PhysioNet/CinC suggests a challenge in 2014 for developing robust heart beat detectors using multimodal signals.

**Methods:**

This article proposes a multimodal data association method that supplements ECG as a primary input signal with blood pressure (BP) and electroencephalogram (EEG) as complementary input signals when input signals are unreliable. If the current signal quality index (SQI) qualifies ECG as a reliable input signal, our method applies QRS detection to ECG and reports heartbeats. Otherwise, the current SQI selects the best supplementary input signal between BP and EEG after evaluating the current SQI of BP. When BP is chosen as a supplementary input signal, our association model between ECG and BP enables us to compute their regular intervals, detect characteristics BP signals, and estimate the locations of the heartbeat. When both ECG and BP are not qualified, our fusion method resorts to the association model between ECG and EEG that allows us to apply an adaptive filter to ECG and EEG, extract the QRS candidates, and report heartbeats.

**Results:**

The proposed method achieved an overall score of 86.26 % for the test data when the input signals are unreliable. Our method outperformed the traditional method, which achieved 79.28 % using QRS detector and BP detector from PhysioNet. Our multimodal signal processing method outperforms the conventional unimodal method of taking ECG signals alone for both training and test data sets.

**Conclusions:**

To detect the heartbeat robustly, we have proposed a novel multimodal data association method of supplementing ECG with a variety of physiological signals and accounting for the patient-specific lag between different pulsatile signals and ECG. Multimodal signal detectors and data-fusion approaches such as those proposed in this article can reduce false alarms and improve patient monitoring.

## Introduction

### Backgrounds

Monitoring heartbeats by analyzing electrocardiogram (ECG) signals offers non-invasive, low-cost, and immediate interpretation of cardiac activity. The QRS complex in an ECG signal is a typical and notable target to detect heartbeats, as the amplitude of the R-peak is at the highest level on the ECG signal waveform. In an ideal environment where the incoming ECG signal is free from noise, a straightforward signal analyzer detects the locations of the QRS complexes in the ECG signal waveform and reports the positions of heart beats. Unfortunately, in the real-world environment where the incoming ECG signal contains various types of noise and artifacts due to improper sensor placement, abrupt body movements, and environmental power sources, QRS detection based analyzers report the heartbeat activity inaccurately. In the worst case, it is difficult to distinguish between the case where the patient is in a serious state and the case where the measurement device has an error.

An accurate interpretation of vital functions is highly required at the digital bedside monitoring and intensive care unit (ICU). With the patient monitoring, robust estimation of patient’s condition from contiguous physiological measurements is critical because medical staff must react immediately to monitoring results [1]. However, the accuracy of monitoring can be lowered due to a variety of technical problems including low-voltage leads, motion artifacts, falsely low oxygen saturation readings in hypothermic patients, and a wet flow sensor of a ventilator [2]. Excessive false alarms during the monitoring slow down response times and decrease the quality of care [3].

In the last few decades, a number of algorithms have been developed for estimation of patient conditions and reporting cardiac activities. Most methods are focused on detecting QRS complexes and reporting heartbeats [4–7]. Time domain analyses detect the QRS complex straightforwardly, but they miss periodic information. On the other hand, frequency-domain analyses assess a variety of frequency components of an ECG signal containing noise. The quantitative and qualitative evaluations with public databases show that gqrs method achieves the best results in terms of the sensitivity level and positive predictive values [8, 9].

### PhysioNet/CinC challenge 2014

While the high accuracy QRS complex detection is currently available, the reliable detection of heartbeats remains an open challenge, as exemplified by the PhysioNet/CinC challenge 2014 [10]. The main goal of the challenge is to help the development and comparisons of robust methods for detecting heartbeats in long-term multi-channel physiological measurements [ECG, blood pressure (BP), stroke volume (SV), photoplethysmogram(PPG), electroencephalogram (EEG), electrooculagram (EOG)]. This challenge is motivated by the limitation of unimodal QRS detectors operating on ECG signals when the incoming ECG input signal is either inaccurate or missing. The challenge encourages researchers to explore the question of to what extent an examination of other physiological signals, such as the BP, EEG, and respiration, can help improve the detection of beats associated with heart activity. For instance, in most subjects, the observed relationships between the respiration and heart rate can be used to model the heart rate, and together with nearby information derived from ECG or other cardiac signals, these models can predict heartbeat locations from respiratory signals.

The PhysioNet/CinC challenge 2014 exhibits a variety of algorithms: the RS slope [11], an autocorrelation similarity matrix [12], the removal of spurious beat detections using non-ECG signals [13], symbolic aggregation approximation [14], a derivative-based peak finder of BP and PPG [15], multimodal lead switching [16], integer multiplier digital filters [17], wavelet transform, dynamic thresholds and moving windows [18], and correlations with an averaged shape [19]. These algorithms were structured in three steps: (i) detecting QRS complexes, (ii) extracting alternative solutions after analyzing multimodal physiological signals, and (iii) combining candidate solutions. Most of the top entries used pulsatile information, which led to a small advantage of up to 5 % over the gqrs method operating on a single ECG signal. If the breathing is unstable or the pulsatile signals are difficult to analyze, we need a complementary model from non-pulsatile information.

The aims of this research are: (i) to determine the optimal timing for the QRS detection method using the signal quality index (SQI), (ii) to propose a complementary model using non-pulsatile information when ECG and pulsatile signals are not available, and (iii) to evaluate the performance of the proposed method relative to those methods presented in the PhysioNet challenge 2014 and its follow-up entries.

## Materials and data

The challenge consisted of two data sets: a public training data set and hidden test data set [20]. The public training data set contains 100 records which are sampled at 250 samples per second. The hidden test data set contains 300 records which have various lengths and sampling rates between 120 and 1000 samples per second. The test data set is hidden and available for the testing of the performance of the detection of heartbeats.

PhysioNet provided challenge data in the form of ten-minute multi parameter records of human adults. Each record contains four to eight physiological signals, and each signal is one of ECG, electromyography (EMG),EEG, electrooculogram (EOG), BP, photoplethysmogram (PPG), respiration (Resp), stroke volume (SV), and the oxygen saturation (SO2) signals. Each record begins with an ECG signal value and is followed by a variety of simultaneously recorded physiologic signal values. A set of reference beat annotations for each record was produced by expert opinions about the locations of the observed QRS complexes in the ECG signals. We classify the physiological signals into two groups according to pulsatile and non-pulsatile characteristics with the cardiac activity. The pulsatile group consists of BP, Resp, SV, and SO2. The non-pulsatile group consists of EMG, EEG, and EOG.

**Fig. 1:**
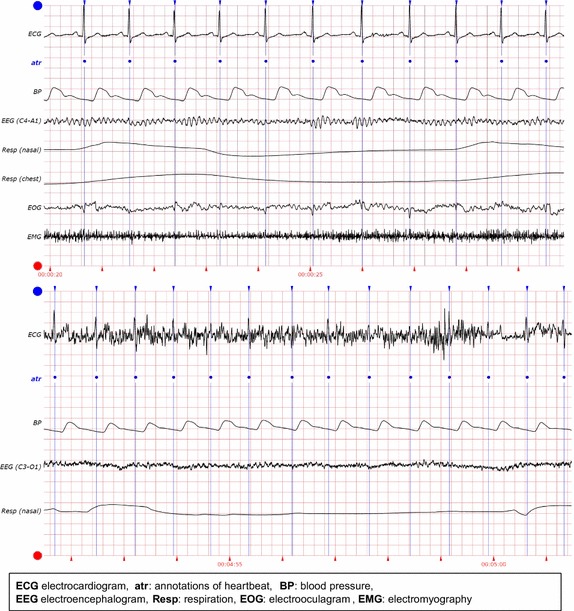
Example of records. Two examples of records in data sets: (*upper*) physiologic signals in a normal condition, (*below*) physiologic signals in a noisy condition

Figure 1 visualizes time series of example data sets for heartbeat detection under normal and noisy condition. The first signal in both conditions is the ECG signal. Under the normal condition, the ECG signal is stable and the QRS complexes and heartbeats are clearly identifiable. Under the noisy condition, the ECG signal is exceptionally noisy for a QRS detector. On the other hand, the ECG does not affect the quality of other physiologic signals (e.g., BP).

## Methods

### Proposed method

**Fig. 2:**
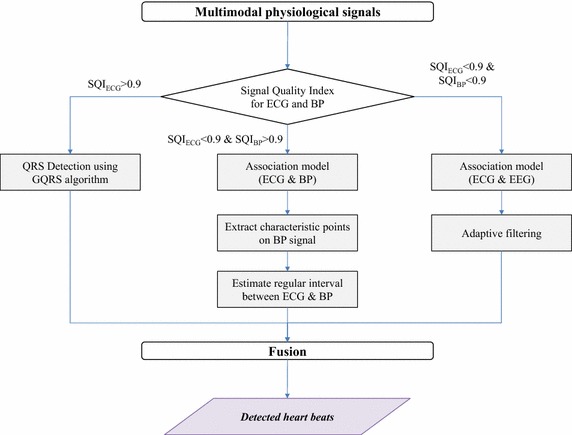
Workflow diagram of the proposed method for multimodal physiological signals

The proposed method for the robust detection of heartbeats is described in Fig. 2. The architecture is based upon novel signal quality metrics and integration of association models from the ECG, BP, and EEG. To evaluate the quality of the incoming signals, signal quality metrics for the ECG and BP waveforms are calculated using several SQI metrics [21]. When the SQI metrics qualify the ECG signal as a reliable one, the QRS complex detector operates on the stable ECG signal waveform and searches for heartbeats. Otherwise, the SQI metrics chooses the best supplementary input signal out of the BP and EEG signals. In case of supplementing ECG with BP, regular intervals between BP and ECG signal are estimated from the detected QRS complexes and characteristic BP signals directly associated with cardiac activity. When the SQI metrics disqualify both ECG and BP, we resort to the association model between ECG and EEG that relates EEG with cardiac activity and apply an adaptive filter to the EEG signal to locate heartbeats. Finally, the fusion component selects one of the outgoing results from these three sub-modules for the candidate locations of heartbeats.

### Signal quality index of ECG and BP signals

Signal quality index evaluates the incoming signals and selects which source signals to analyze to report heartbeats. When a signal quality index indicates that the quality of incoming ECG signal is below a certain threshold, the QRS detector does not report heartbeat precisely. We devised signal quality index metrics for the ECG and BP signals: $SQI_{ECG}$ and $SQI_{BP}$.

$SQI_{ECG}$ combines the relative-power based and momentum-based SQI proposed in previous research for various window length and rhythm cases [21, 22]. *pSQI* refers to the relative power in the QRS complex: $\int _{ 5Hz }^{ 15Hz }{ P(f)df } /\int _{ 5Hz }^{ 40Hz }{ P(f)df }$. *kSQI* is the momentum-based SQI as the fourth moment (kurtosis) of the distribution. The kurtosis of an ECG segment was calculated for 10 s epochs. From the predefined criteria, the *kSQI* value is defined with binary value (0 and 1) [21]. Both *pSQI* and *kSQI* are already normalized from 0 to 1 and we have calculated the average of two SQI values as SQI for ECG. This averaged SQI is termed $SQI_{ECG}$.

$SQI_{BP}$ evaluates the quality of the BP signal using an open-source algorithm [23] which flags a signal of poor quality if derived parameters from a blood pressure wave are not in reasonable physiological ranges. Only the pressure ranges and the average derivative for a cycle (a subset of the original SQI parameters) were checked for validity. This SQI is henceforth termed $SQI_{BP}$.

$SQI_{ECG}$ and $SQI_{BP}$ control which input signals to process in reporting heatbeats. We set the switching threshold to be 0.9. If SQI for ECG is more than the switching threshold (i.e., $SQI_{ECG} \ge 0.9$), the ECG was used. Otherwise, if SQI for BP is more than the switching threshold (i.e., $SQI_{ECG}<0.9$ and $SQI_{BP}\ge 0.9$), the BP was used. Finally, if both ECG and BP signals are unreliable, our method resorts to the EEG signal.

### QRS detection

QRS detectors operating on an unreliable ECG signal are useful since the percentage of clean segments on the ECG signal is greater than the percentage of contaminated segments in long-term measurements. To find the best SQR detector as a component of our multimodal analysis architecture, we evaluated and compared the sqrs, wqrs and gqrs detection methods as provided from PhysioNet as open sources for training and hidden data. The sqrs detection method is based on slope detection using predefined filters for five slopes (P, Q, R, S, and T waves). In wqrs analyses of ECG signals, QRS onsets and J-points are detected using nonlinearly scaled ECG curve length feature. The gqrs detection method is based on R-peak intervals, and has been optimized for sensitivity. Table 1 shows that the best QRS detection was achieved by the *gqrs* method. The sqrs method shows the worst sensitivity (Se) and positive predictive value (PPV). The wqrs and gqrs methods show competent results on training data, scoring 99 %. However, we still have room to improve, as can be seen in the results with the hidden data. Consequently, the gqrs method was used with clean ECG signals.

**Table 1:** Performance of QRS detection methods used for training and the hidden data of the PhysioNet challenge dataset

QRS Detector	Se (%)		PPV (%)		Overall (%)
	Gross	Average	Gross	Average	
$\texttt {sqrs}_{training}$	20.42	21.19	*99.97*	*99.98*	60.39
$\texttt {wqrs}_{training}$	99.85	99.86	92.00	95.37	96.77
$\texttt {gqrs}_{training}$	*99.94*	*99.95*	99.25	99.33	*99.61*
$\texttt {sqrs}_{hidden}$	62.24	62.98	63.44	68.11	64.19
$\texttt {wqrs}_{hidden}$	*88.90*	*89.82*	73.08	76.26	82.01
$\texttt {gqrs}_{hidden}$	88.49	88.22	*82.91*	*84.42*	*86.01*

### Association model between ECG and BP signals

When the ECG signal is unreliable, and the BP signal is reliable, our method processes the BP signal and locates heartbeats based on an association model for estimating regular intervals between ECG and BP signals. We extracted characteristic points from ECG and BP signals for the estimation. The locations of the assumed QRS candidates in ECG signals are used as the characteristic points. In addition, the locations of the local maximum and minimum points in the BP signals are used as the characteristic points. According to observations of the waveform and the characteristic points of the ECG and BP signals, the signals show a regular interval for each consecutive heartbeat caused by blood circulation from cardiac activity. The QRS candidates are followed by the characteristic points of the BP signal.

**Fig. 3:**
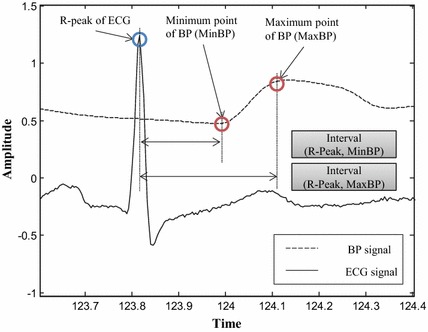
Interval between ECG and BP signals Intervals between the R-peak of ECG signal and characteristic points of the BP signals

Figure 3 shows the characteristic points and intervals from ECG and BP signals. The characteristic points are the maximum and minimum points of BP signals after each R-peak of the ECG signal. While observing the training data, we realized that the minimum point of the BP signals (*MinBP*) guarantees more steady intervals than the maximum points of the BP signals (*MaxBP*). Using the R-peak and *MinBP*, we define the local intervals for each heartbeat:

1 $$\begin{aligned} Interval_{{local_i}} = t_{MinBP_i} - t_{{R-peak}_i} \end{aligned}$$

where ${R-peak}_i$ is the ith detected R-peak using QRS Detection of the ECG signal and $MinBP_i$ is the maximum point of the BP signals, which is followed by the ith R-peak.

From empirical observing the training data, proportion of unreliable ECG signal is less than 5 %. Thus, we selected 50 consecutive local intervals from the first beat of each record. local intervals are used to estimate a regular interval, as follow:

2 $$\begin{aligned} Interval_{regular} = average(Interval_{{local_i}}) \end{aligned}$$

3 $$\begin{aligned} Heartbeat_{estimation} = t_{MinBP_i} - Interval_{regular} \end{aligned}$$

**Fig. 4:**
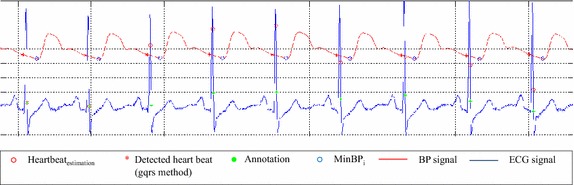
Consecutive intervals between ECG and BP signal etected heartbeats from regular interval between the ECG signal and BP signals

As shown in Fig 4, we used the regular interval and extracted the characteristic points of the BP signals to detect heartbeats. In comparison with annotations and results by gqrs method, our proposed method successfully detected the heartbeats using association model from BP signal.

The main advantage of the association model, compared to the single QRS detector which is based on the R-peak interval, is that the reliable heartbeat detection can be extracted from even a very noisy ECG signal with a relatively high sensitivity and specificity. However, the disadvantage of the association approach is that it cannot proceed when the sufficient phyiological information are not provided. There are constraints in the measurement environment.

### Association model between ECG and EEG signals

When both ECG and BP signals are unreliable, we resort to processing the EEG signal and estimating heartbeats based on association model between ECG and EEG signals. From the perspective of an EEG analysis, it can be seen that the EEG signal is contaminated by QRS complexes which appear as spikes at the same time in the ECG record. The ECG signal increases the difficulty in analyzing with EEG signals and in obtaining clinical information. Therefore, numerous methods have been proposed to correct or remove these artifacts from EEG signals [24–27]. In other words, we can extract cardiac activity including QRS activity or QRS position from a set of EOG, EEG, and EMG signals. Usually, these physiological signals have similar frequency spectra. The adaptive interference cancellation scheme has been proposed to remove or extract signals and interference from polysomnography (PSG) readings [28].

**Fig. 5:**
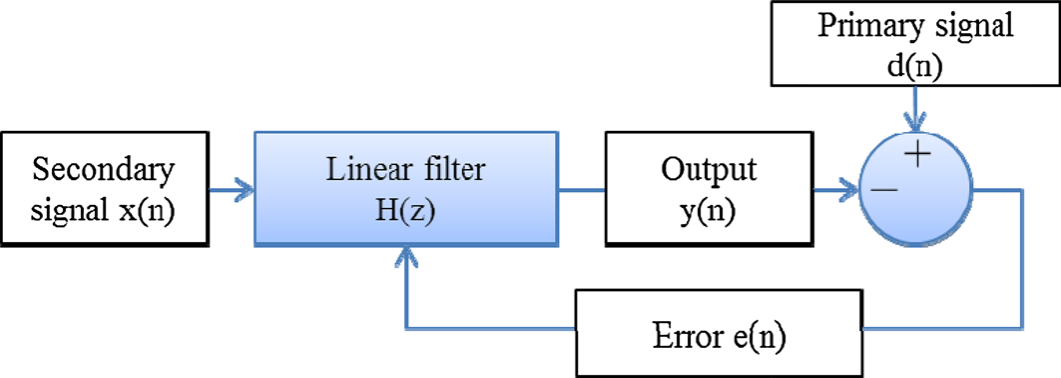
Association model between ECG and EEG signals linear filter H(z) remove cardiac artifacts from EEG signals

The structure of an adaptive filter for EEG signals can be found in the literature [28]. The association model between ECG and EEG signals is given in Fig 5. There is a primary signal *d*(*n*) and a secondary signal *x*(*n*). The linear filter *H*(*z*) produces an output *y*(*n*) which is subtracted from *d*(*n*) to the compute an error *e*(*n*). The main objective of an adaptive filter is to model reliable coefficients of the linear filter *H*(*z*). With successful adaptation to the secondary signal *x*(*n*), the linear filter *H*(*z*) generates output similar *y*(*n*) to that of the secondary signal *x*(*n*). We applied this model with only one filtering layer.

**Fig. 6:**
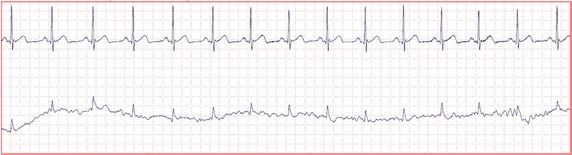
EEG signal with ECG artifact physiological signals in data set. (*upper) ECG signal (below) EEG signal corrupted with ECG artifact*

In this paper, we used the EEG signal as the primary signal *d*(*n*), generated a normal ECG signal for the secondary signal *x*(*n*), and used FIR filters for the linear filter *H*(*z*). *d*(*n*) and *x*(*n*) are EEG and ECG signal from each records in dataset. We concatenated the adaptive filters with different order values of 16, 32, and 64. The result of this process can be found with the output *y*(*n*) as QRS candidates. In Fig 6, it can be observed the QRS complex present in a segment of EEG record. The QRS amplitudes in the ECG are of the order of mV, however in the external EEG thy have been reduced. These artifacts in the EEG records could be used in analysis of heartbeat.

### Multimodal data fusion

**Fig. 7:**
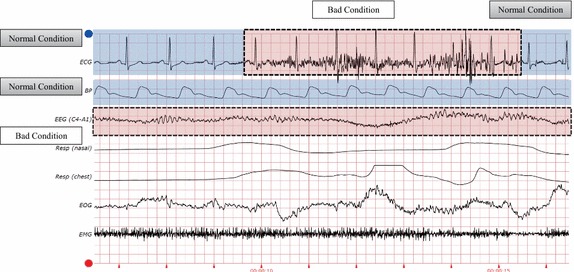
Case of quality levels. Respective quality levels of signals as measured in a comparison with the general shapes from the training data

The most important aspect of the challenge was the development of an intelligent fusion technique for multiple peak detection from different physiological signals. Figure 7 presents the quality of the signals. The blue box denotes a good condition and the red box represents a poor condition. Intuitively, the presence of noise and artifacts will lower the agreement level of a QRS detector. For $SQI_{ECG}$, the agreement level of two SQI measurements in a 10 s window, evaluated every second, was used for the *pSQI* and *kSQI* readings. Two SQI results were recently used successfully in a database with pathological rhythms. The BP signal quality was also evaluated using an open-source algorithm [23]. As mentioned earlier, we utilize a branch point depending on the SQI measurements. The final result is selected by the SQI measurement. If the quality of the ECG signal is sufficient, the result from the QRS detector lends credence to the locations of the heartbeats. From the original assumption, if the quality of ECG signal is inadequate, the BP signal is a representative pulsatile signals, serving as an alternative means of analysis. In our research, we proposed an association model between the ECG and EEG signals as a new alternative analysis method when the quality of both the ECG and BP signals is too low.

## Results

### Evaluation scheme

The proposed method is applied to the every second of every set of hidden test data with 300 records. Our method generates test annotations which mark heartbeat as results. Evaluation application bxb and sumstats which provided by PhysioNet matches our test annotations with reference annotations to count, for each record in the hidden test data, the numbers of correctly-detected beats (true positives, or *TP*), missed beats (false negatives, or *FN*), and detections of non-beats (false positives, or *FP*). $TP_i$, $FP_i$, and $FN_i$ denote the statistics for an individual record. To match a reference annotation, a test annotation must be located within 150 ms of the reference annotation, and must be the nearest test annotation to the reference annotation [10].

For calculation of performance statistics, sensitivity (Se) is the percentage of beats that are true positives, and positive predictivity (+P) is the percentage of detections that are true positives:

4 $$\begin{aligned} Se_{gross} =100\cdot \frac{ TP }{ TP+FN }, \qquad Se_{average}=\frac{100}{n} \cdot \sum _{ i=1 }^{ n }{ \frac{ { TP }_{ i } }{ { TP }_{ i }+{ FN }_{ i } } } \end{aligned}$$

5 $$\begin{aligned} +P_{gross} = 100\cdot \frac{ TP }{ TP+FP }, \qquad +P_{average} = \frac{100}{n} \cdot \sum _{ i=1 }^{ n }{ \frac{ { TP }_{ i } }{ { TP }_{ i }+{ FP }_{ i } } } \end{aligned}$$

The gross statistics are derived from the sum of all *TP*, *FP*, and *FN* over the test data set. The average statistics are the means of the statistics calculated individually for each record of the test data set [10]. Finally, the overall score for each entry was the average of $Se_{gross}$, $Se_{average}$, $+P_{gross}$ and $+P_{average}$.

### Experimental results

**Fig. 8:**
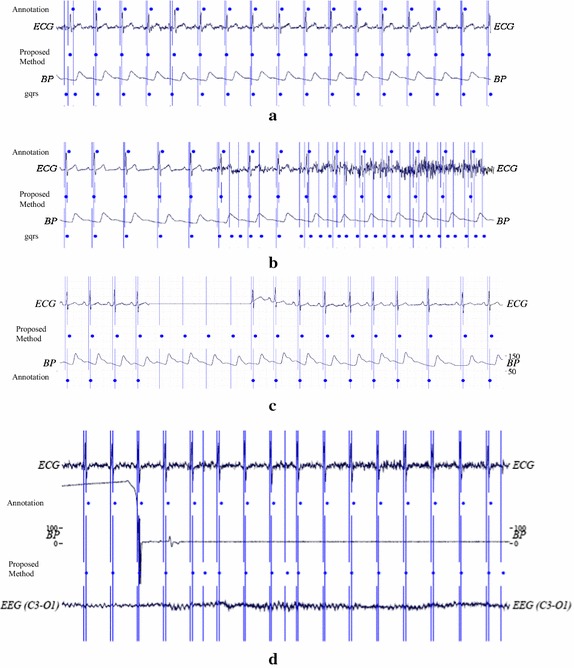
Experimental results. Experimental results of the proposed method: **a** normal condition, **b** unstable condition, **c** missing condition, and **d** noisy ECG and missing BP condition

Figure 8 shows the representative results from the proposed method. In normal conditions where the ECG signal is clean, our method locates heartbeats precisely. Figure 8(b) shows that the existing QRS detection method locates heartbeat inaccurately when the ECG signal contains high-frequency noise. On the other hand, our method automatically switch to gathering additional information from the BP and EEG signals when they are present in the record. In the event of a missing ECG section, our method tracks the heartbeats well with the trained regular inter-beat interval from the estimation module with the ECG and BP signals as shown in Fig 8(c). When both ECG and BP signals are unstable or missing, our proposed method can estimate the location of heartbeats using the result of adaptive filter as shown in Fig 8(d).

Once all records of the training set were processed, and the trained inter-beat intervals were used for the challenge data. The challenge are The datasets from Phase I and Phase II are a subset of the dataset of Phase III. The scores on Phase III are the final results of PhysioNet/CinC challenge 2014. Table 2 shows the evaluated overall scores for the challenge. The top score for the challenge was 87.9 %, achieved by an earlier method [16]. The proposed method achieved a score of 86.26 %.

**Table 2:** Official rankings

Phase I		Phase II		Phase III	
Vollmer	93.2	De Cooman	86.2	Johnson	87.9
Pangerc	89.2	Vollmer	86.0	Soo-Kng	86.7
Johannesen	88.9	Pangerc	85.9	De Cooman	86.6
Ding	88.9	Plesinger	85.0	Gieraltowski	86.4
Soo-Kng	88.7	Johnson	84.6	Vollmer	86.2
gqrs	89.8	gqrs	85.7	gqrs	84.5

The overall scores for the sample entry (gqrs) are shown for comparison

As can be seen from the Table 3, we have performed systematic comparison of accuracy with four different conditions: ECG alone, ECG with BP, ECG with EEG, and all three signals. The BP signal shows a major role in the experiment results. The experiment result shows that the EEG part is contributed on the little portion of experiment result. We conclude from a reason that the contribution of EEG part were less effective. The signals included in the validation data set are varied from the each record. ECG and BP signals are included in the training and validation data set. However, EEG signal is only included with ten records from the revealed validation data set with 100 records.

**Table 3:** Systematic comparison of the accuracy with three different conditions: ECG alone, ECG + BP, and ECG + EEG

Signals	Overall scores (%)	
	Training data	Hidden test data
ECG alone	99.61	84.50
ECG + BP	99.82	85.98
ECG + EEG	99.77	84.78
ECG + BP + EEG	99.97	86.26

## Conclusions

This article presents a framework for a heartbeat detection method for Computing in Cardiology 2014. The final results indicate that the proposed method works well in various noisy scenarios and conditions. Overall, the use of EEG signals was shown to be beneficial with both noisy ECG and BP signals. Through reverse-engineering, our method removed the ECG artifacts from EEG signals, and we successfully found clues for detecting heartbeats in the EEG signals. Unlike other methods, we measured the SQI of ECG and BP signals to detect which signals are reliable. This leads to an efficient branch point for removing redundant calculations.

In future work, we plan to process various types of BP signals, such as the arterial blood pressure and pulmonary arterial pressure. Also, the EMG signals captured on the lower body can be useful to analyze cardiac activity when the patient’s upper body is tossing and turning. Furthermore, the proposed method can be improved with a standardized signal quality index (SQI) and by adaptive tuning of the criteria of the SQI measurements. Also, the association model of BP signals can be improved by integration with a peak detector for BP signals.

## Abbreviations

BP: blood pressure
ECG: electrocardiogram
EEG: electroencephalogram
EMG: electromyogram
EOG: electroculogram
ICU: intensive care unit
PPG: photoplethysmogram
RESP: respiration
SO2: oxygen saturation
SQI: signal quality index
SV: stroke volume
